# Deep learning enables the atomic structure determination of the Fanconi Anemia core complex from cryoEM

**DOI:** 10.1107/S2052252520009306

**Published:** 2020-08-20

**Authors:** Daniel P. Farrell, Ivan Anishchenko, Shabih Shakeel, Anna Lauko, Lori A. Passmore, David Baker, Frank DiMaio

**Affiliations:** a Department of Biochemistry, University of Washington, Seattle, WA 98105, USA; b Institute for Protein Design, University of Washington, Seattle, WA 98105, USA; c MRC Laboratory of Molecular Biology, Cambridge, United Kingdom

**Keywords:** Fanconi anemia core complex, cryoEM, distance predictions, deep learning

## Abstract

Cryo-electron microscopy of protein complexes often leads to moderate resolution maps (4–8 Å), with visible secondary-structure elements but poorly resolved loops, making model building challenging. In the absence of high-resolution structures of homologues, only coarse-grained structural features are typically inferred from these maps, and it is often impossible to assign specific regions of density to individual protein subunits. This paper describes a new method for overcoming these difficulties that integrates predicted residue distance distributions from a deep-learned convolutional neural network, computational protein folding using *Rosetta*, and automated EM-map-guided complex assembly. We apply this method to a 4.6 Å resolution cryoEM map of Fanconi Anemia core complex (FAcc), an E3 ubiquitin ligase required for DNA interstrand crosslink repair, which was previously challenging to interpret as it comprises 6557 residues, only 1897 of which are covered by homology models. In the published model built from this map, only 387 residues could be assigned to the specific subunits with confidence. By building and placing into density 42 deep-learning-guided models containing 4795 residues not included in the previously published structure, we are able to determine an almost-complete atomic model of FAcc, in which 5182 of the 6557 residues were placed. The resulting model is consistent with previously published biochemical data, and facilitates interpretation of disease-related mutational data. We anticipate that our approach will be broadly useful for cryoEM structure determination of large complexes containing many subunits for which there are no homologues of known structure.

## Abstract 2

This paper describes a method for determining an atomic model of a protein complex using moderate-resolution cryoEM data and distance predictions from deep learning.

## 1. Introduction   

With the advent of direct electron detectors and advances in image-processing software, there has been an influx of large protein complex structures determined by cryoelectron microscopy (cryoEM). These technologies allow the structural characterization of protein assemblies that have eluded X-ray crystallography, and have led to maps with resolutions that allow atomic models to be built directly (3.3–4.6 Å or better; Cheng & Walz, 2009 ▸; Hryc *et al.*, 2011 ▸) or lower subnanometre resolutions (∼5–9 Å) that can be interpreted by the fitting of existing models. CryoEM data are noisy and structure determination requires a large number of particle images to be averaged together. This averaging, when combined with complications such as image misclassification, highly heterogeneous samples or a limited number of sample views, typically limits the resolutions that can be attained (Lyumkis, 2019 ▸). This makes map interpretation difficult, and has necessitated the development of a number of tools for model building and refinement into such cryoEM maps (Bonomi *et al.*, 2019 ▸; Chen *et al.*, 2016 ▸; Segura *et al.*, 2016 ▸; Terashi & Kihara, 2018 ▸; Terwilliger *et al.*, 2018 ▸; van Zundert *et al.*, 2015 ▸).

In the absence of homologous structural information, cryoEM maps at intermediate resolutions are often largely ‘uninterpretable’; that is, while secondary structures may be identified and domains can often be roughly segmented, atomic-level information may not be accurately inferred (Gatsogiannis *et al.*, 2013 ▸; Janssen *et al.*, 2015 ▸; Kube *et al.*, 2014 ▸; Stuttfeld *et al.*, 2018 ▸). At best, a combination of secondary-structure placement and sequence-based secondary-structure prediction can lead to low-resolution complete or partial backbone-trace models (Cheng *et al.*, 2010 ▸; Snijder *et al.*, 2017 ▸). Furthermore, while computational tools exist for modeling into maps at these resolutions (Bonomi *et al.*, 2019 ▸; Kovacs *et al.*, 2018 ▸; Segura *et al.*, 2016 ▸; van Zundert *et al.*, 2015 ▸; Webb *et al.*, 2018 ▸), no tool is capable of inferring such structures *de novo*. Finally, while co-evolution information can provide valuable structural information (Kim *et al.*, 2014 ▸; Nugent & Jones, 2012 ▸; Ovchinnikov *et al.*, 2014 ▸), the limited availability of large numbers of sequences restricts the applicability of the method, although it has been used in the interpretation of some cryoEM structures (Klink *et al.*, 2020 ▸; Park, Lacourse *et al.*, 2018 ▸; Schoebel *et al.*, 2017 ▸).

In this manuscript, we take advantage of recent advances in protein structure prediction which employ deep convolutional neural networks to predict protein contacts or pairwise distances from multiple sequence alignments (Kandathil *et al.*, 2019 ▸; Nugent & Jones, 2012 ▸; Senior *et al.*, 2020 ▸; Xu, 2019 ▸; Yang *et al.*, 2020 ▸; Zheng *et al.*, 2019 ▸). We combine predictions from *trRosetta* (Yang *et al.*, 2020 ▸), which uses a deep residual-convolutional neural network to predict both distance and orientation between all pairs of residues in a protein, and a fast model-building protocol that utilizes the results from the network to constraint folding. We then dock models generated using this approach into cryo-EM maps. The experimental EM data and deep-learning-based structure predictions are synergistic: the deep-learned predictions serve the same role as high-resolution structures of homologues, informing the topology of individual domains and making the search space manageable, while the EM data, addresses two weaknesses in contact-guided prediction: it validates the accuracy of contact-guided predictions, and secondly, it provides information on the quaternary structure of complexes.

We illustrate the effectiveness of this approach by building an atomistic model of the *Gallus gallus* (chicken) Fanconi anemia core complex (FAcc), guided by a recently published heterogeneous 4.6 Å resolution single-particle cryoEM reconstruction and cross-linking mass-spectrometry data. In previous work, crystal structures of FANCF, FANCE and FANCL were docked and secondary-structural elements were placed into the map (Shakeel *et al.*, 2019 ▸). In contrast, here we are able to generate an atomistic model for nearly all of the complex. This method overcomes the limitations of direct interpretation of the cryoEM map, including a lack of recognizable homology to proteins of known structure for the majority of the subunits, and the relatively low resolution of substantial portions of the complex. The novel structural information provided by *trRosetta*-predicted distance distributions enables accurate topology-level predictions for domains and subunits with no recognizable homology. By combining these *trRosetta* predictions (and *Rosetta* density-guided modeling tools; Wang *et al.*, 2016 ▸) with subnanometre-resolution cryoEM data, we are able to infer a nearly complete FAcc model, providing key insights into the function and organization of this complex.

## 2. Methods   

### 2.1. Composite-map generation   

The cryoEM map used for all computations (and displayed in all figures) is a composite map generated from three individual focused refinements. The EMDB IDs of these maps are EMD-10293 (bottom), EMD-10292 (middle) and EMD-10291 (top). The maps were combined by first aligning each map to the ‘bottom’ map in *UCSF Chimera* (Pettersen *et al.*, 2004 ▸) using the ‘fit into density’ tool and resampling using ‘vop resample’. Next, using a custom script, the ‘bottom’ map was normalized to density values between 0 and 1, and histogram matching was used to remap the density distributions of the ‘middle’ and ‘top’ maps to that of the ‘bottom’ map. Finally, a weighted average of the three maps was computed, in which the weight of the contribution of each map to the composite map was proportional to the density value in each map at a given point.

The local resolution plots in Supplementary Fig. S6(*b*) were estimated using *ResMap* (Kucukelbir *et al.*, 2014 ▸) by using the aligned maps generated in the previous step and their respective half maps.

### 2.2. Subunit multiple sequence-alignment generations   

In order to model the subunits of FAcc, we first generated multiple sequence alignments (MSAs) for every subunit of FAcc using a two-step procedure. In the first stage, four rounds of iterative *HHblits* (version 3.0.3; Steinegger *et al.*, 2019 ▸) searches against the Uniclust30 database (August 2018 version) with gradually relaxed *E*-value cutoffs (10^−80^, 10^−70^, 10^−60^, 10^−40^ and 10^−20^) were used to generate an initial alignment. The resulting alignment was then converted to an HMM profile and additional sequences were collected by a single run of *hmmsearch* (version 3.1b2; Eddy, 1998 ▸) against an extensive custom sequence database as described in Wu *et al.* (2020 ▸); a bit-score threshold of 0.2 × (protein length) was used to select significant hits. The composite MSAs were filtered with *hhfilter* at 99% sequence-identity and 50% coverage cutoffs.

### 2.3. *trRosetta* domain model building   

We used *trRosetta* to predict the structures of the following components: FANCA, FANCB, FANCC, FANCE, FANCF, FANCG, FANCL and Fanconi anemia core complex associated protein 100 (FAAP100). *trRosetta* model building is a two-step process: in the initial step a deep residual convolutional neural network is used to generate inter-residue distance and orientation predictions, and in the second step these predictions are used to model a protein of interest (Yang *et al.*, 2020 ▸). The MSAs were used as inputs to the neural network, which generates residue pair distance distributions in addition to orientation information between all residue pairs. These predictions are then used as input to a custom *Rosetta*-based folding protocol. This protocol works by randomly setting backbone torsions and utilizing random subsets of the predictions as restraints for a centroid (*Rosetta*’s reduced residue representation) torsional quasi-Newton-based energy minimization (*MinMover*). For each domain, 150 centroid models are generated and each model is then refined with the *Rosetta* full-atom FastRelax protocol. The results from this refinement are used to sort the models based on the REF2015 score function, and the top three models are selected and manually inspected. For all domains except the CC domains and FAAP100 α/β+CtH we observed a well converged structure, and representative structures from this modeling are shown in Fig. 3(*a*).

The original *trRosetta* pipeline was unable to generate converged models for the sequence between the β-propeller regions and the β-sandwich regions of FAAP100 and FANCB and for the sequence of FAAP100 α/β+CtH, so we employed a modified version of the network which, in addition to the MSA, also used information on the top 50 putative structural homologs as identified by *HHsearch* against the PDB100 database of templates. *HHsearch* hits were converted into 2D network inputs by extracting pairwise distances and orientations from the structure of the template for the matched positions only. Additionally, the positional (1D) similarity and confidence scores provided by *HHsearch* as well as backbone torsions were used; we tiled them in both axes of the 2D inputs and stacked with them, producing the resulting 2D feature matrix. Features for all unmatched positions were set to zero. Templates were first processed independently by one round of 2D convolutions and were then merged together into a single 2D feature matrix using a pixel-wise attention mechanism. This processed feature matrix was then concatenated with the features extracted from the MSA as in the original *trRosetta* network; the architecture of the upstream part of the network remained unchanged. For the CC domains this improved the quality of the models for the β-propellers as well as the models for the extended helices C-terminal to the β-propellers. For FAAP100 α/β+CtH we modeled FAAP100 CC+βsand+α/β+CtH with this modified version and found strong convergence for all of the domains. The coiled-coil domains of FANCB and FAAP, and FAAP100 α/β and CtH, were manually extracted for use in the next stages. The results from this modified version of *trRosetta* are shown in Supplementary Fig. S2(*b*).

### 2.4. Inferring domain boundaries   

To infer domain boundaries, we used the MSAs as initial guidelines by adding cut points at residues with poor alignment coverage. Using these domain definitions, we then performed structural modeling of the domains and used these models to manually split the sequence further based on the observed convergence and trimmed away floppy regions. The following domain boundaries were determined: FANCA, 71–260, 251–500, 500–651, 1011–1210; FANCB, 1–235, 1–370, 231–365, 441–660, 441–780, 466–626, 651–770, 665–755; FANCC, 1–175, 1–335, 176–335, 331–570; FANCE, 1–150, 261–520; FANCF, 1–130, 121–350, 142–350; FANCG, 1–175, 1–320, 181–320, 201–435, 204–315, 321–648, 350–564; FANCL, 1–100, 2–91, 101–205, 101–300, 104–373, 191–300, 301–373; FAAP100, 1–200, 1–300, 28–442, 186–480, 301–480, 491–615, 491–820, 510–609, 711–820, 717–820.

Then, based on the availability of homologous structures in these regions, either *RosettaCM* (if homologous structures were available; Song *et al.*, 2013 ▸) or *trRosetta* (if homologous structures were not available; Yang *et al.*, 2020 ▸) were used to generate models for each domain.

### 2.5. *RosettaCM* domain model building   

We modeled FANCE_CtH_, FANCF_HR_, FANCG, FANCL_ELF+URD+RING_ and FAAP100_βprop_ using *RosettaCM* (Song *et al.*, 2013 ▸). The following templates were used for each subunit. FANCE_CtH_: PDB entry 2ilr (chain *A*). FANCF_HR_: PDB entry 2iqc (chain *A*). FANCG: PDB entries 6eou (chain *A*), 2xpi (chain *A*), 3hym (chain *J*), 3cvp (chain *A*), 4rg9 (chain *A*), 5dse (chain *A*), 3fp2 (chain *A*), 5orq (chain *A*), 5i9f (chain *A*), 4g1t (chain *B*), 3ieg (chain *A*), 2y4t (chain *A*), 5aio (chain *A*), 4pjr (chain *A*), 1fch (chain *B*), 4zlh (chain *B*), 2gw1 (chain *A*), 6c9m (chain *C*), 3u4t (chain *A*), 4buj (chain *B*). FANCL_ELF+URD+RING_: PDB entries 3k1l (chain *B*), 4zdt (chain *A*), 4ccg (chain *Y*), 1vyx (chain *A*), 5o6c (chain *A*), 2d8s (chain *A*). (The resulting models were segmented further before docking.) FAAP100_βprop_: PDB entries 4ggc (chain *A*), 5opt (chain *p*), 5xyi (chain *g*), 6chg (chain *A*), 5oql (chain *F*), 2pbi (chain *D*), 1r5m (chain *A*), 6eoj (chain *D*), 6f9n (chain *B*), 5m89 (chain *B*), 3odt (chain *B*), 5a31 (chain *R*), 5m23 (chain *A*), 5kdo (chain *B*).

For each of the above, 200 models were generated using the command line
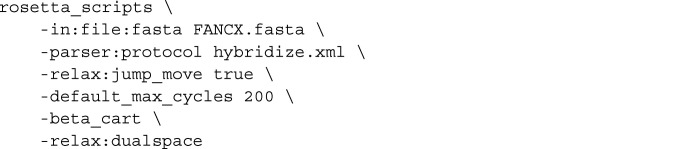


The input XML file (hybridize.xml) is shown below:
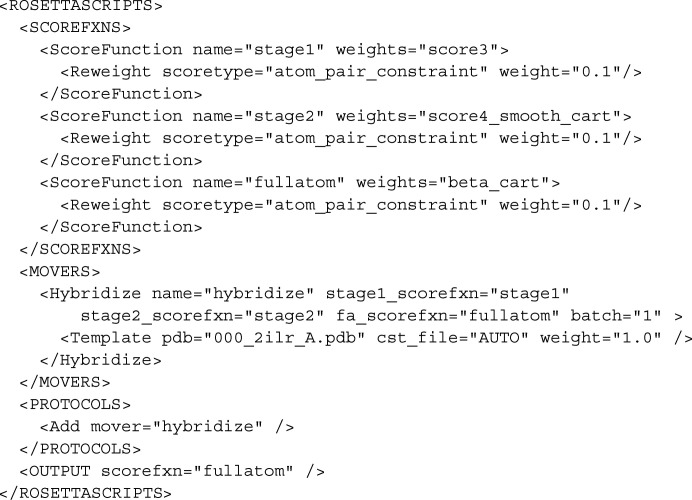


### 2.6. Docking of domain models into density   

A new *Rosetta* tool, *dock_pdb_into_density*, and a wrapper script (dgdp.py, for density-guided domain placement) were used for the initial assembly of models. Briefly, *dock_pdb_into_density* uses an FFT-accelerated six-dimensional search to find the rigid-body placements of a molecule that maximize the overlap between model and map. For all domains that were modeled, we began by docking the top three models into density using *dock_pdb_into_density*. This method carries out FFT convolutions in rotational space, explicitly enumerating over translations; the method identified 50 000 points with high density (and >2 Å apart). For each domain, all solutions were combined, and the top 1000 were filtered and rigid-body minimized in *Rosetta* using a masked correlation function (DiMaio *et al.*, 2009 ▸). After minimization, the results were filtered for redundancy (using an 11 Å r.m.s. cutoff) and the top 200 solutions were selected.

The following command line carries out the procedure for FANCC:
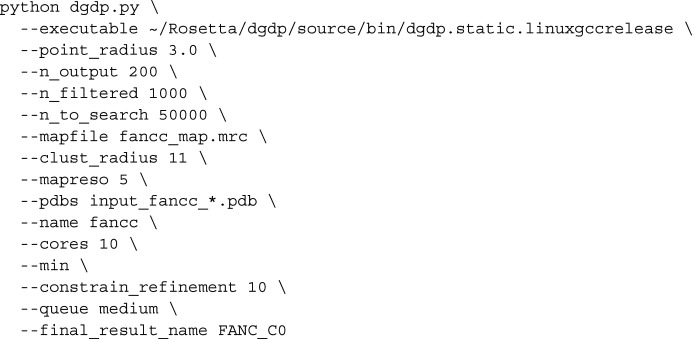


The typical CPU usage for docking one model is highly dependent on the size of the density map and the number of residues in the model, but for FAcc we generally see docking take a total CPU time of 5.5 h. However this is highly parallelizable, and by using the Python Dask library (Dask Development Team, 2016 ▸) with ample computing resources the total time taken can be reduced significantly (54 min with ten CPUs).

### 2.7. Docked domain assembly   

Given the docked domains from the previous section, we used a modified version of the Monte Carlo simulated-annealing (MC-SA) sampling protocol described in Wang *et al.* (2016 ▸) to build a model of the complex. Briefly, the protocol uses the top 200 placements for each model from our docking protocol, in addition to the cross-linking data, in order to determine a set of domain placements that are most consistent with all available data. This MC-SA domain assembly assigns a placement or ‘not found’ to each domain to account for the possibility that either all of our predicted models are incorrect or that the domains are correct but not present in the map. Consistency is measured through the function (where *d_N_* is all domains)
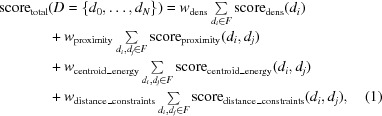
where score_dens_ measures the fit of the selected domains to the density and the other terms assess interactions between all domains. The term score_proximity_ validates that when two domains are part of the same peptide chain and not overlapping they are placed within a distance that is closable by a subsequently built peptide linker. The score_centroid_energy_ term is *Rosetta*’s centroid energy score term, which is a coarse-grained representation that is used to verify the quality of domain–domain interfaces, as well as to screen for clashing placements. The centroid energy between two domains is evaluated by using *Rosetta* to combine the two domains into a single system (Pose), evaluating the energy of the system and then spatially separating the two domains and again evaluating the energy of the system. The former is subtracted by the latter, and this is used as the unweighted score_centroid_energy_. Finally, the score_distance_constraint_ term serves as a way to incorporate experimental data such as cross-linking mass-spectrometry data, and assesses the satisfaction of these constraints. The inter-domain geometry terms are assessed as
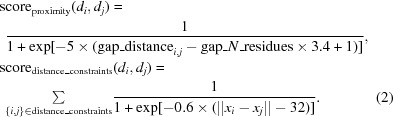



Weights were determined by fitting on a training set with synthetic 10 Å resolution cryoEM data, and the weights used were *w*_dens_ = 260, *w*_distance_constraint_ = 30 000, *w*_centroid_energy_ = 150 and *w*_proximity_ = 1000.

Using the scoring function above, we evaluated the consistency of the results from docking for all domain–domain pairs. Prior to score-function evaluation a custom pairwise interface-optimization protocol was applied: domains are slid along an axis through the center of mass of each domain to be in contact, but not clashing, with each other, moving no more than 5 Å. If after this domains are still clashing (defined as a *Rosetta* vdw score of >1500), we remove all clashing residues (*Rosetta* vdw score of >3) with (i) no secondary structure and (ii) surface exposure (less than ten C^α^ atoms within 12 Å) and rescore. This is then followed by breaking both domains into subdomains (using a re-implementation of *DDomainParse* in *Rosetta*; Zhou *et al.*, 2007 ▸) and rigid-body minimizing these domains with respect to the energy function above.

Once all pairs of domains have been refined and their refined inter-domain energies have been computed, MC-SA sampling was carried out. Each MC-SA move reassigns one domain to either another placement or to ‘no placement.’ We carried out 200 000 steps of MC-SA sampling, ending at a temperature of *kT* = 1. 50 000 independent trajectories were carried out and the top ten scoring assignments were assessed for convergence. Convergence was assessed by manually inspecting the ten domain assignments for domains that are present in a majority of the models. This process was applied iteratively; after each round of assembly, domains that converged in location were locked into place, where convergence was assessed, the density occupied by converged domains was removed from the density map, and unassigned domains were redocked and used as inputs for the next round.

In the case of FAcc, the iterative process progressed as follows. After the first round, FANCB_α/β_, FANCC, FANCE_NtD_, FANCF, six helices of FANCG (residues 204–315) and FANCL were found to have converged and were locked into place. After the second round, eight helices of FANCG (residues 350–564) were locked into place. After this round, the density associated with the two β-propellers was segmented out, and both the FANCB and FAAP100 β-propellers were docked into this segmented density and were used as inputs for the next round. During the third round, the β-propellers of FANCB and FAAP100 and the two coiled coils of FANCB and FAAP100 were both frozen into place. In the fourth round, we found converged placement of FANCB_βsand_. After this round, utilizing the remaining models and density, we manually docked the FAAP100_βsand_, FAAP100_α/β_ and FAAP100_CtH_ domains into the density.

### 2.8. Structure finalization   

In order to finalize the structure, all of the placed domains from the previous step were combined and linked together with *RosettaCM* (Song *et al.*, 2013 ▸). There were five areas that required directed rebuilding with *Rosetta*: the FANCB β-propeller, the FANCB loop between the CC helix and the β-sandwich, the FANCB C-terminal helix, FANCG and the FAAP100_α/β+CtH_ domain.

The FANCB β-propeller as solved by *trRosetta* was very similar to the density, but the spacing between each propeller blade was off by a significant enough margin to make it difficult for *RosettaCM* to properly minimize into the density. Therefore, we ran an automatic domain-splitting script and used *dock_pdb_into_density* to place subdomains of the propeller into the β-propeller density assembled using the protocol described above.

The FANCB helix-to-β-sandwich loop required excessive sampling to build owing to its length (41 residues) and lack of density. The density around this area was segmented, and *Iterative Hybridize* (Park, Ovchinnikov *et al.*, 2018 ▸) was run with the initial amount of structures generated being 5000, followed by four rounds each generating 100 structures.

The FANCB extended helix built with *trRosetta* was added, using *UCSF Chimera*’s ‘fit into map’ tool, after the α/β domain of FANCB had been placed. This was performed because of the unambiguous density leading from the α/β domain extending into helical density.

For FANCG, assembly placed only two domains, corresponding to residues 204–315 and 350–564. The remaining structure was built in the following way. Firstly, the N-terminal domain (1–204) was well converged in *trRosetta* and was manually docked into the map by aligning it with overlapping residues in one of the placed domains (residues 200–230 overlapped between the two). The same process was carried out with the C-terminal domain (residues 565–648), where the overlapping residues used were residues 551–562. These placements were validated by manually inspecting the fit to density.

The FAAP100_α/β_ domain posed a particularly difficult problem owing to low local resolution and poor connectivity (this domain is preceded by a long unstructured loop). Owing to these ambiguities, FAAP100_CtH_ models had to be manually aligned to the density (using *UCSF Chimera*’s ‘fit into map’ tool). Full-length *trRosetta* models were used as a reference for placement.

Finally, after refining the structure with *RosettaCM*, we applied fragment-based structural refinement (Wang *et al.*, 2016 ▸) and selected the top-scoring model as our final model.

### 2.9. Data accessibility   

All methods are available in *Rosetta* releases after 2020.12. The model has been deposited in PDB-Dev (https://pdb-dev.wwpdb.org/) as entry PDBDEV_00000055. The cross-linking data that were used as distance constraints during this process are located in the PRIDE database (https://www.ebi.ac.uk/pride/) with accession code PXD014282.

## 3. Results   

**Figure 1:**
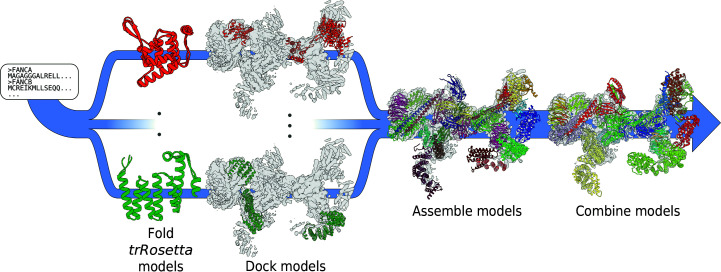
An overview of the workflow for modeling FAcc. Initially, multiple sequence alignments (MSAs) of all protein sequences are generated, the sequences are segmented into domains using the MSAs and the individual domains are folded using *trRosetta*. These domains are individually docked into the cryoEM density. Monte Carlo sampling finds the domain assignment that is maximally consistent with the experimental data (electron density, XL-MS *etc.*, Fig. S5). Finally, linkers between domains are sampled and the entire structure is refined.

A full description of our methodology is described below and in Section 2. Briefly, Fig. 1 ▸ illustrates how we determine complex structures using *trRosetta* models. The protocol is a five-stage process where we first generate multiple sequence alignments (MSAs) for the target proteins and use these to manually segment sequences into domains. Secondly, *trRosetta* is used to build models corresponding to these domains. Thirdly, using *dock_into_density* in *Rosetta*, we search the cryoEM reconstruction for the best-matching placements of each domain model. In the fourth step, we take all docked results in addition to cross-linking mass-spectrometry (XL-MS) data, and using Monte Carlo sampling of domain assignments in density we find the arrangement of (and choice of) domain models that maximizes the agreement with the cryoEM and XL-MS data. Finally, using *RosettaCM* we rebuild the connections between domains and refine the entire complex against the cryoEM map.

**Figure 2:**
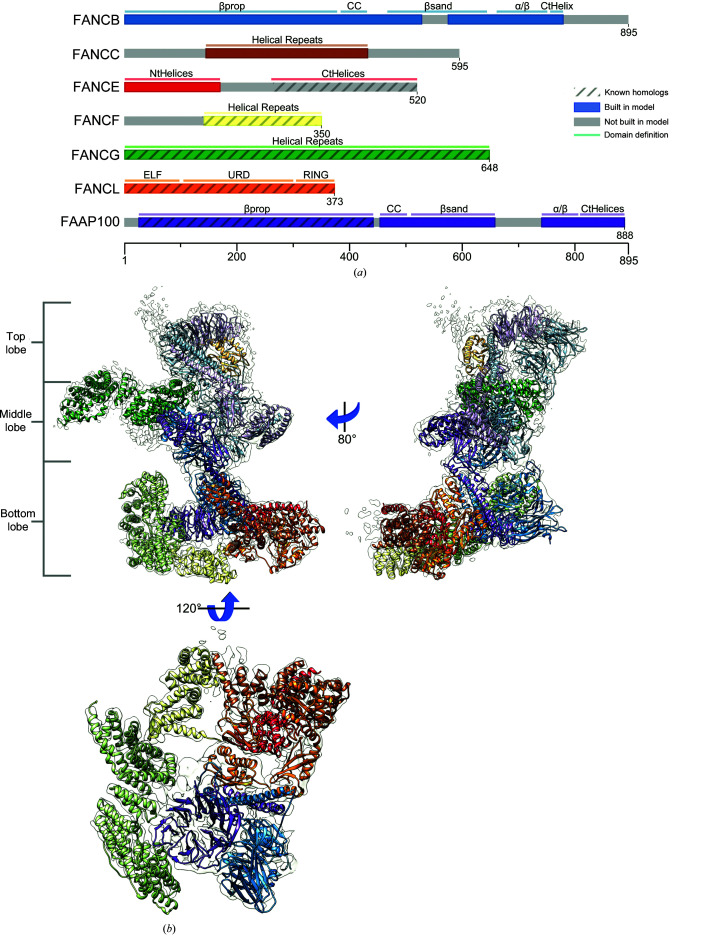
An overview of FAcc. (*a*) Domain organization of the seven subunits of FAcc. Based on our modeling, we find that the complex consists of 18 domains, indicated with narrow bars. FANCB and FAAP100 have the same domain organization, with a β-propeller (βprop) followed by a long coiled coil (CC), a β-­sandwich (βsand) and then an α/β domain, finally followed by a C-­terminal helical region. FANCC, FANCF and FANCG are all comprised of a single helical-repeat domain, while we find FANCE to have two separated helical-repeat domains (one N-terminal and one C-­terminal). Finally, FANCL is organized as an ELF domain followed by a URD domain and then lastly a RING domain. Also indicated is the availability of known structures or homologous proteins throughout the modeling process with striations. Domains with known structures or available homologous proteins used include the C-terminal helices of FANCE, the helical repeats of FANCF, all of FANCG and FANCL, and the β-propeller of FAAP100. (*b*) Three views of the complete model of FAcc as determined by our modeling protocol. Colors are matched to the diagram in (*a*), with those that have multiple copies (FANCB, FANCG and FAAP100) having different shades of the coloring. The orientations of the top, middle and bottom lobes are indicated.

We illustrate the power of *trRosetta* predictions by applying this approach to build an atomic model into the recently determined cryoEM reconstruction of the Fanconi anemia core complex (FAcc; Shakeel *et al.*, 2019 ▸). These data were obtained from a fully recombinant complex after the overexpression of eight protein subunits (FANCA, FANCB, FANCC, FANCE, FANCF, FANCG, FANCL and FAAP100) in insect cells. A 3D reconstruction at an overall resolution of 4.6 Å and cross-linking mass-spectrometry data were obtained. Secondary structure elements were previously identified within the majority of the cryoEM map and fitted with homology models [Fig. 2 ▸(*a*)]. Using cross-linking, native and hydrogen–deuterium exchange mass spectrometry, as well as EM of purified subcomplexes, the general locations of all components were identified, except FANCA. However, in this previous work, residue assignments were confidently determined only for FANCL. To gain further insight into the molecular mechanisms of FAcc, atomic models of all subunits are required.

Using *trRosetta*-predicted distance distributions, we were able to determine a complete sequence assignment of the full FAcc [Fig. 2 ▸(*b*)], encompassing 5182 residues out of an expected total of 6154 residues, or 84% of the sequence, with very little unexplained density (Supplementary Fig. S1). Modeling did not make use of the domain assignments or the backbone trace of the prior work. Our model validates many of the putative subunit assignments from the prior study (with minor differences) and provides residue-level detail of subunit locations and interactions. The next several sections describe the modeling process, followed by an analysis of our final model.

### 3.1. Fold *trRosetta* models   

Our protocol uses multiple sequence alignments (MSAs) for individual proteins as the input to a deep residual convolutional network which predicts the relative distances and orientations of all residue pairs in the protein. These predictions are applied to a restrained minimization using a *Rosetta* model-building protocol. For FAcc, MSAs were generated for every chain without known homologous structures [homology models were available for portions of FANCE, FANCF, FANCL and FAAP100; see Fig. 2 ▸(*a*)]. Although homologous structures to FANCG also exist, there was significant structural variability within the family, and therefore we modeled FANCG with *trRosetta* in addition to building homology models.

**Figure 3:**
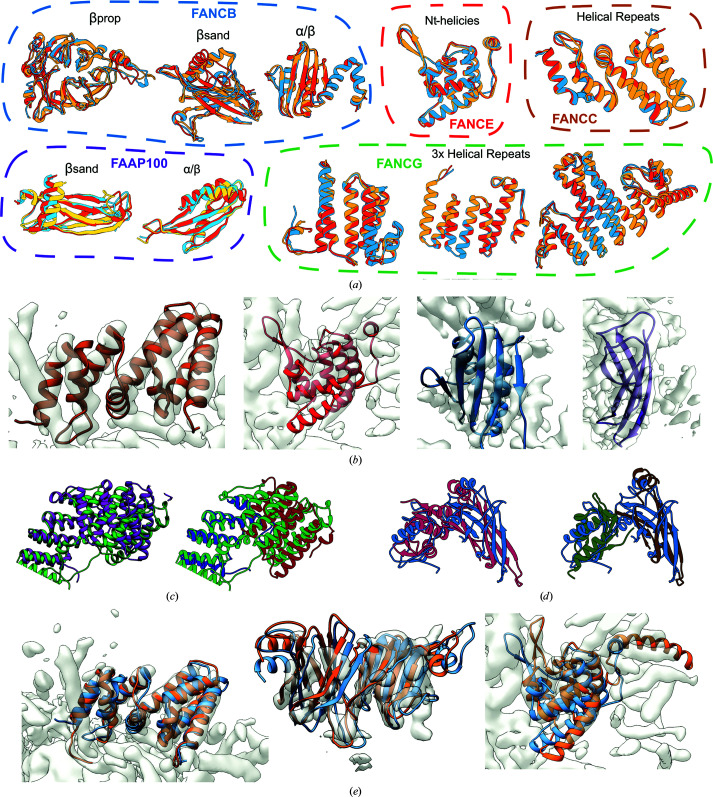
An overview of *trRosetta*-predicted domains. (*a*) The top three models from *trRosetta* for ten representative domains indicate a tight convergence of modeling. The identity of the domains follows the coloring in Fig. 2 ▸(*a*). Domains from FANCB, FANCE and FANCC are shown in the top row, while those from FAAP100 and FANCG are shown in the bottom row. (*b*) Several examples of *trRosetta* models docked into density before refinement, showing the role that the map plays in the validation and selection of models. From left to right [the colors match those in Fig. 2 ▸(*a*)]: the helical repeats of FANCC, the N-terminal repeats of FANCE, the α/β domain of FANCB and the β-sandwich of FAAP100. (*c*, *d*) Two examples illustrating the importance of domain segmentation when docking *trRosetta*-generated models. (*c*) The *trRosetta* model of FANCG (magenta) poorly matches the final structure (green); segmenting this model into two domains (red and blue) shows a much better match, as the individual domain structures are accurate, even though their relative orientation is not. (*d*) Similarly, a *trRosetta* prediction of the FANCB β-sandwich–α/β domain (pink) is dissimilar from the final structure (blue); splitting it into domains (brown and green) shows good overall agreement. (*e*) *trRosetta* models (blue) generally fit the map well, although some refinement was necessary to maximize agreement with the density (orange).

From the MSAs, domains were manually parsed (see Section 2), and models were built using *trRosetta* (in regions with no known homologs) or comparative modeling (in regions with known homologs). Modeling yielded converged structures for almost all domains [Fig. 3 ▸(*a*)], with typical maximal r.m.s.d.s over the top models of 2–4 Å. Several of the domains that showed poor convergence (two of the domains in FANCB and two of the domains in FAAP100) still contained subregions (‘converged cores’) with small deviations (2–4 Å) between models; for these cases, unconverged or poorly packed segments of the models were manually trimmed. Three of the domains (the coiled-coil domains of FANCB and FAAP100 and the α/β and CTH domains of FAAP100) were poorly converged with no ‘converged core’; a modified version of *trRosetta* (unpublished work) in which structural information on distant homologues was used as input to the neural network led to well converged models. In total, *trRosetta* was able to build all 42 attempted domains [Supplementary Fig. S2(*a*)], which were used in the subsequent stages of the model-building protocol.

### 3.2. Assembling domains into cryoEM density   

While we found FANCL, FANCF and FANCE_NtD_ straightforward to manually place into the map, ambiguity in the placement of the other subunits necessitated a more robust automated assembly procedure. Initially, the top five models for each domain were docked using an FFT-accelerated 6D search of the map. A modified version of the MC-SA sampling protocol described in Wang *et al.* (2016 ▸) was then used to identify the nonclashing placement of models that maximized the overall fit of the complex model to the density. This MC-SA domain assembly assigns a placement or ‘not found’ to each domain to account for the possibility that either all of our predicted models are incorrect or that domains are correct but not present in the map. In this way, the map serves not only to orient domains but also as validation for the *trRosetta* predictions. Some examples of model validation with the map are shown in Fig. 3 ▸(*b*). Two examples of incorrect predictions (subsequently fixed by splitting models into two domains) are shown in Figs. 3 ▸(*c*) and 3 ▸(*d*). For several domains (the aforementioned coiled-coil domains of FANCB and FAAP100 and the FAAP100 α/β and CtH domains), manual docking was necessary.

In order to model FAcc in its entirety, this Monte Carlo simulated-annealing assembly process was applied iteratively: in each round, the converged domains from the previous round were frozen, and all unassigned domains were redocked and reassembled. Convergence was assessed by manually inspecting the ten domain assignments with the best overall agreement with the density and the XL-MS data. Once the iterative process had converged (after five rounds), with the vast majority of the density occupied, the connections between domains were built and refined in the context of the cryoEM density with *RosettaCM* (Song *et al.*, 2013 ▸). Additionally, the placed domains were individually inspected and poorly placed segments were also rebuilt in *RosettaCM*.

When refining the final assembled model we found that most *trRosetta* models were quite accurate, often requiring only modest (<6 Å r.m.s.d.) modifications throughout the refinement process [Fig. 2 ▸(*e*)]. Only one placed domain required significant movement: the β-propeller domain of FANCB. To refine this domain, the model was automatically segmented into subdomains (see Section 2) and was redocked and assembled using the same Monte Carlo procedure before refinement. A comparison between the initial and final structures of the β-propeller after this protocol is shown in Supplementary Video S1.

Finally, for FANCG, a repeat protein for which homologous structures were available, we additionally used *trRosetta* for modeling, as predicting changes in repeat geometries can prove challenging. As Supplementary Fig. S3 shows, *trRosetta* yielded models that contained two long adjacent helices between residues 416 and 491, while homology modeling generated models which contained four shorter helices. In assembly, both *trRosetta* and homology models were considered, and we found that the *trRosetta* models led to a much better agreement between the model and the map. In contrast, in previous work homology modeling was used for FANCG resulting in the placement of only ∼280 residues into the map (Shakeel *et al.*, 2019 ▸).

### 3.3. Analysis of the final model   

Using our protocol, we were able to build and assign 5182 residues (out of 6557 in the full complex); in previous work only 337 residues were assigned. Still, the protein and domain identities assigned previously were largely consistent with the models obtained with this new method: we found similar placements of FANCB, FANCF, FAAP100 and FANCL, as well as of one of the two copies of FANCG. While we were unable to identify any density associated with FANCA, *trRosetta* provided well converged models [Supplementary Fig. S2(*a*)]. Combining these models with the cross-linking data, we speculate that a region of unassigned density in the middle of the complex corresponds to FANCA (Supplementary Fig. S4). However, owing to the poor quality and incompleteness of the density in this region, we were not able to confidently dock the model into the map.

Our final model reveals that the ‘bottom lobe’ [Fig. 2 ▸(*b*)] contains FANCB_βprop_, FANCL, FANCE, FANCF, FANCG and FAAP100_βprop_ [using the domain terminology of Fig. 2 ▸(*a*)]. In contrast, in previous work FANCC and FANCE were identified within a region of density that we assign to FANCG. The ‘middle lobe’ of our model consists of two copies of FANCB_βsand+α/β+CtH_, a second copy of FANCG and two copies of FAAP100_βsand+α/β+CtH_, all of which are consistent with the previously proposed domain assignment (Shakeel *et al.*, 2019 ▸). Finally, the ‘top lobe’ was found to contain a second copy of FANCB_βprop_, FANCL_ELF_ and a second copy of FAAP100_βprop_. This also is consistent with the hypothesized model from the prior work. Finally, both the top and bottom lobes were connected to the middle lobe through a FANCB and FAAP100 intermolecular coiled coil. Thus, in addition to validating much of the domain assignment of previous work, our new model now provides accurate positioning of all protein residues.

### 3.4. Model validation   

One potential source of model validation arises from the cross-linking data. However, as these data were used in domain assembly, they do not serve as independent validation data. As a measure of confidence, we can still use these data by analyzing the gap between the satisfied cross-links in our model and the number satisfied by the second-best domain arrangement. In our final model, we see good agreement between the cross-links and the model (Fig. S5) (144 of 188 in total; most of the 834 cross-links in the full data set involve FANCA, which is not present in our model). Of the inter-domain cross-links, 39 of 59 (66%) are satisfied to a CA–CA distance of 30 Å, which is regarded as an acceptable distance given the usage of the BS3 cross-linker (Merkley *et al.*, 2014 ▸). Freezing the unambiguously placed domains and redocking the remaining potentially ambiguous domains (see Section 2) finds that a second-best arrangement, which replaces FANCC with the Ct-helices of FANCE, satisfies only 33 of 63 (52%) inter-domain crosslinks. This loss of inter-domain cross-link satisfaction provides fairly strong confidence in our final model. Further analysis of the unsatisfied cross-links reveals that most of the unsatisfied cross-links (14 of 19) occur between the C-terminus (residues 103 onwards) of FANCL. Our model suggests that one of the two copies of FANCL in the complex has a disordered C-terminal domain, strongly suggesting that most of the unsatisfied cross-links come from this disordered (and possibly dynamic) region.

One particularly strong criteria for model validation is the agreement of the maps with individual domain models. The *trRosetta* models of individual domains were predicted without using density data at all, so rigid-body fitting of these domains into density can be seen as ‘independent validation’. Aside from domains that exhibit internal symmetry or pseudo-symmetry (FANCB_βprop_ and FAAP100_βprop_), we found that the *trRosetta* predictions all matched with real-space correlations of 0.72 or better (FANCC, 0.82; FANCE_NtD_, 0.75; FAAP100_βsand_, 0.75; FAAP100_α/β_, 0.72), while the second-best solution (the best ‘wrong’ solution) has a correlation that is worse by at least 0.05 in all cases. Subjectively, these second-best, incorrect placements look significantly worse. For our placed domains, this gap between the best and second-best solutions is quite large and strongly suggests that these domains are unlikely to match this well by random chance.

The overall agreement between the refined model and map is consistent with what we would expect given the resolution of the data. We were able to assess the quality of our model by segmenting it against the three individual focused classification maps (used to generate the composite map used in modeling). We find that the model–map correlation for the bottom and middle reconstructions crosses an FSC of 0.5 at about 7.2 Å, while the top reconstruction crosses an FSC of 0.5 at about 7.1 Å. The overall model–map FSC curves [Supplementary Fig. S6(*a*)] show that the model–map agreements are worse at higher resolutions for the ‘bottom’ reconstruction than the other two, which is consistent with local resolution estimates [Supplementary Fig. S6(*b*)].

**Figure 4:**
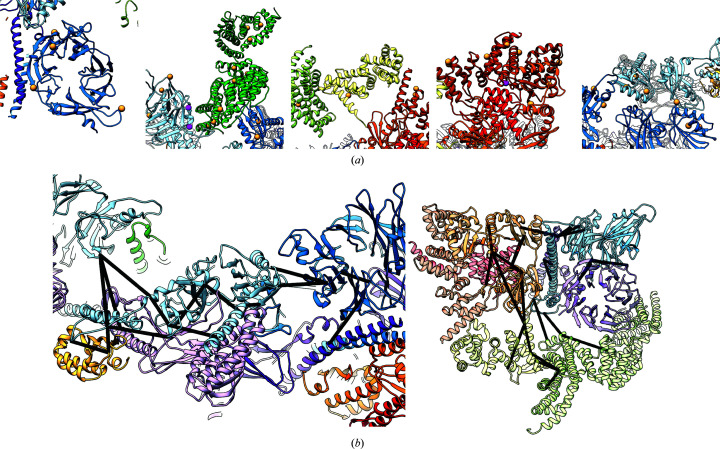
Model validation by mutational and cross-linking data. (*a*) 30 nonbenign human mutations mapped to our model of FAcc. All interface mutations are marked with magenta spheres; non-interface mutations are marked with tan spheres. (*b*) Close-up renderings of cross-links throughout the FAcc model. Black lines indicate cross-links that are satisfied (<30 Å) by the final refined structure. Representatives from each cross-link cluster are shown for the middle lobe (left) and the bottom lobe (right).

Additionally, we can validate models by mapping human mutation data onto the final structure. Using the Fanconi Anemia Mutation Database (http://www2.rockefeller.edu/fanconi/), we identified 30 mutations that were not identified as benign throughout the complex. While most (22) of these are in the core of protein subunits (and are likely to destabilize individual subunits), we identified four (of the remaining eight) at protein–protein interfaces in our model of the FAcc complex. Mutations of FANCB residues 230 and 236 would appear to disturb the interface between FANCB_βprop_ and FANCG_HR_, while a mutation at FANCB residue 336 would disturb the interface between FANCB_βprop_ and FAAP100_βprop_. Additionally, a mutation of FANCC residue 295 would be likely to disturb the interface between FANCC and FANCE. All interface mutations are marked as magenta spheres in Fig. 4 ▸(*a*), while non-interface mutations are marked with tan-colored spheres.

## 4. Discussion   

Here we report a new computational method for determining atomistic models of protein complexes, guided by a subnanometre cryoEM map and cross-linking mass spectrometry data. Using distance distributions predicted from deep residual neural networks, we built accurate models of 42 domains of the FAcc, obviating the necessity for homologous high-resolution structures for interpretation of intermediate resolution maps. This provides a complete picture of the full FAcc, while previous efforts had resulted in atomic models for only three subunits (FANCL, FANCE and FANCF) in the map. The strong agreement between *RosettaTR*-predicted models and density (not used in prediction) provides validation of our predictions, as does the model’s consistency with biochemical data, including cross-linking mass spectrometry and mutational studies. Our all-atom model provides molecular insight into the underlying mechanisms of previously reported disease-causing mutations, and illustrates the potential of combining intermediate resolution cryoEM density and cutting-edge de novo structure prediction.

The challenges faced when determining a model of the FAcc are not unique (Chou *et al.*, 2019 ▸; Kim *et al.*, 2018 ▸). As microscopists pursue larger, more difficult, and more dynamic complexes, we will need more computational techniques that are able to build models of subnanometre resolution data with little to no homologous structure information available. While tools have been developed for integrative modelling of structures into subnanometre resolution density, all of these tools require either the existence of homologous structures for domains, or are necessarily low-resolution ‘domain level’ models. Previous attempts to model FAcc resulted in only 387 residues being assigned to the cryoEM data, while the methods described in this paper – making use of 42 deep-learning guided domain predictions, and a protocol able to infer their arrangement – were able to increase the number of assigned residues to 5182.

Our approach shows that, while maps at these resolutions are not of sufficient quality to build models by direct chain tracing, the resolution is sufficient to assess the tertiary structure and accuracy of predicted models. In the absence of high-resolution homologous structures, the method is able to determine structures to an atomic level of detail. In addition to cryoEM data, we have recently shown that a similar approach can be applied to solve low-resolution crystal structure data where traditional molecular replacement techniques were unsuccessful (Bhargava *et al.*, 2020 ▸). We expect that the modeling power of *trRosetta* and related techniques will continue to improve in the future as the number of known sequences increases, coupled with improvements in deep-learning methodologies. We anticipate that this combined approach will be an important tool for determining atomic models of protein complexes, particularly when combined with low-resolution data sources, enabling accurate protein complex structure determination without the requirement of high resolution data.

## Supplementary Material

Supplementary Figures. DOI: 10.1107/S2052252520009306/eh5009sup1.pdf

Supplementary Video S1. Comparison between the FANCB β-propeller before and after refinement. The video shows that although the trRosetta model correctly predicted the β-propeller topology, the spacing between propellers required significant refinement in order to properly fit the electron density. DOI: 10.1107/S2052252520009306/eh5009sup2.mp4
