# Supplementary material for: Deep learning enables the atomic structure determination of the Fanconi Anemia core complex from cryoEM
Source: IUCrJ. 2020 Aug 20;7(Pt 5):881–92. doi: 10.1107/S2052252520009306 (PMC7467173; doi:10.1107/S2052252520009306)
Supplement: Supplementary file 1 [file m-07-00881-sup1.pdf]

IUCrJ

Volume 7 (2020)

Supporting information for article:

Deep learning enables the atomic structure determination of the Fanconi Anemia core complex from cryoEM

Daniel P. Farrell, Ivan Anishchenko, Shabih Shakeel, Anna Lauko, Lori A. Passmore, David Baker and Frank DiMaio

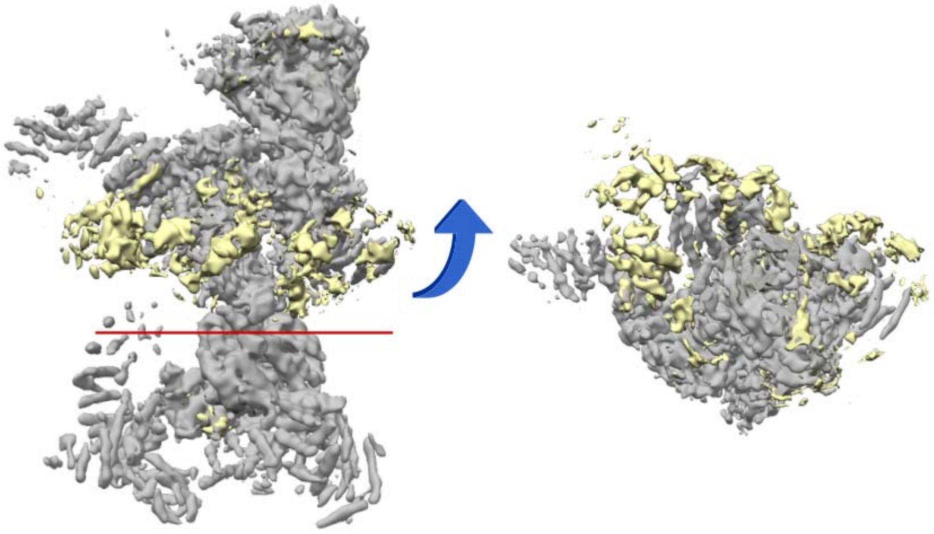

Figure S1 An overlay of the density map used to build the FAcc complex (Grey), with the density not within 3.5 Å of the model highlighted in yellow. We were unable to build into the yellow density due to the low resolution, and lack of continuity between regions.

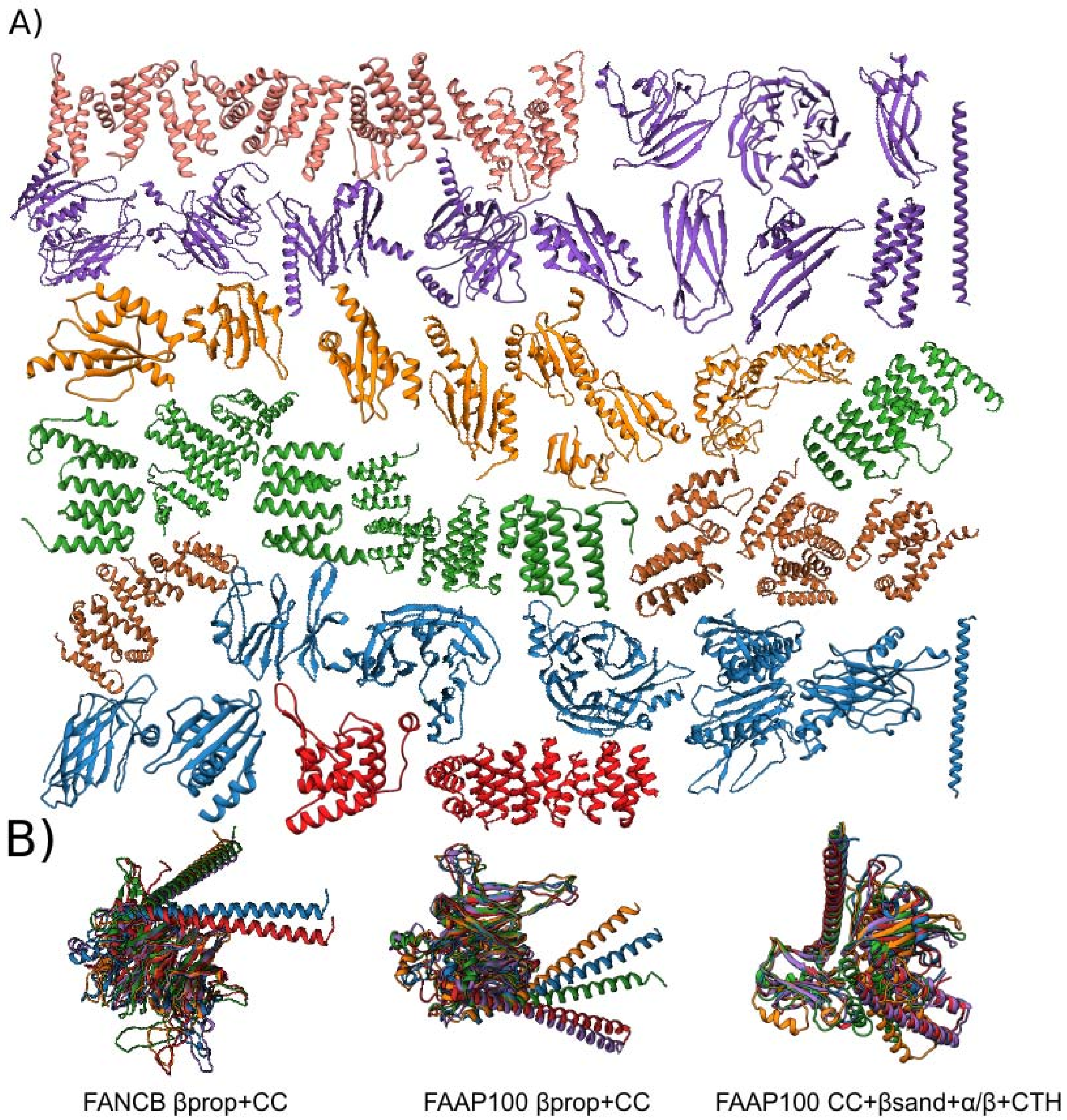

Figure S2 Top scoring models for all of the domains built in order to build the FAcc. A) All domains that were individually docked into the density. Colors match Figure 2A. B) All models built by the novel implementation of *trRosetta* that uses distant homology information in order to improve prediction accuracy. In these three cases, the original implementation of *trRosetta* was unable to generate well converged models for the combinations shown.

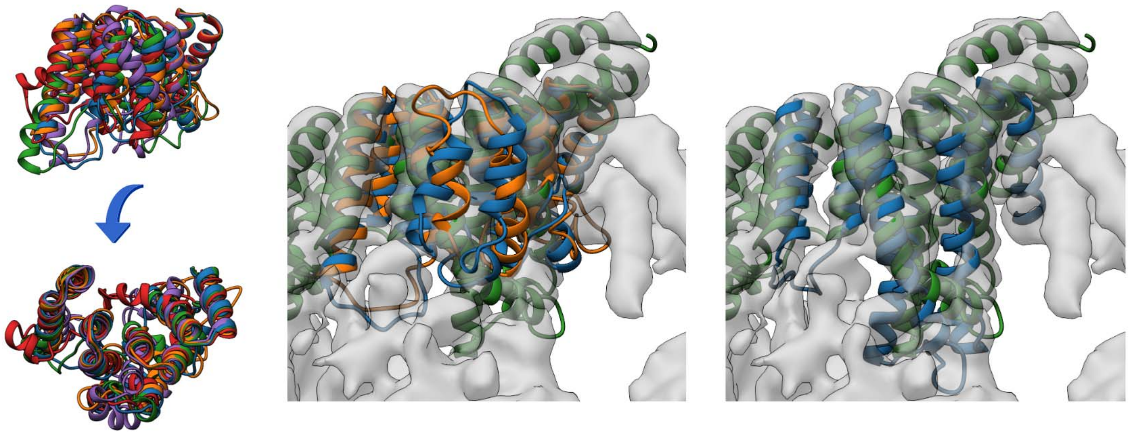

Figure S3 Top 5 scoring ensemble from *RosettaCM* from FANCG (left), with the top 2 models overlaid on the final structure of FANCG (middle). The right shows the model made by *trRosetta*, which fits the density significantly better than the *RosettaCM* models

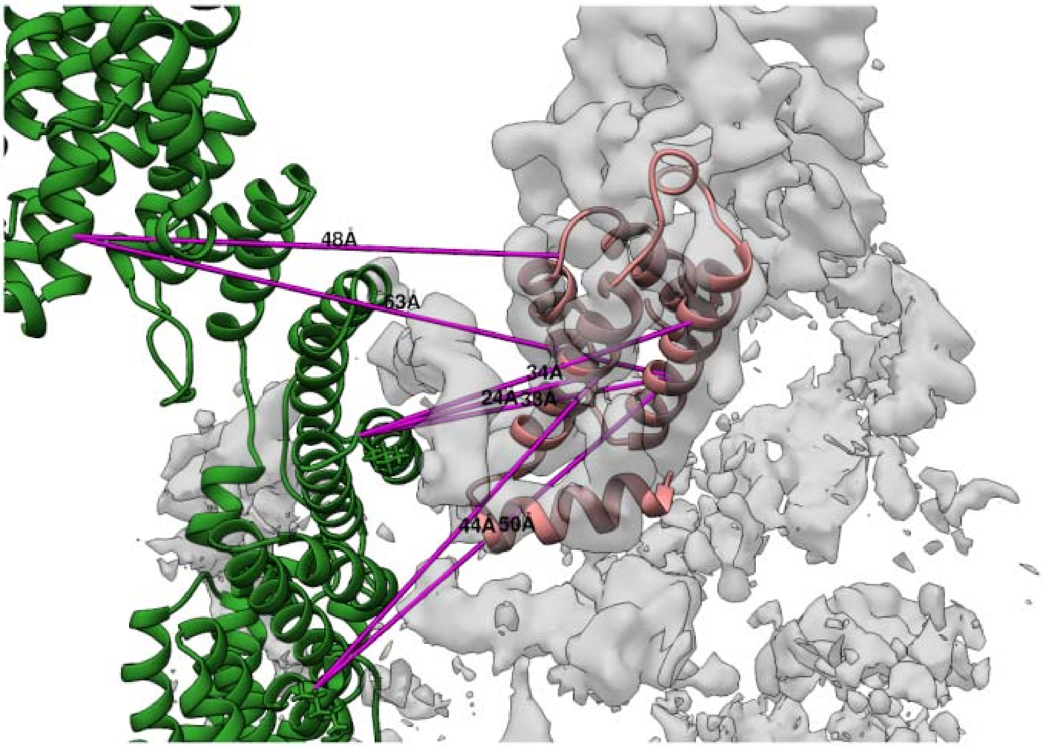

Figure S4 A speculative location for the N-terminal domain of FANCA (salmon) docked into the electron density with crosslinks to FANCG (green) represented as purple lines. We hypothesize, based on the *trRosetta* models fit to the sparse electron density, and crosslink satisfaction that the N-terminal domain of FANCA rests at or near this location.

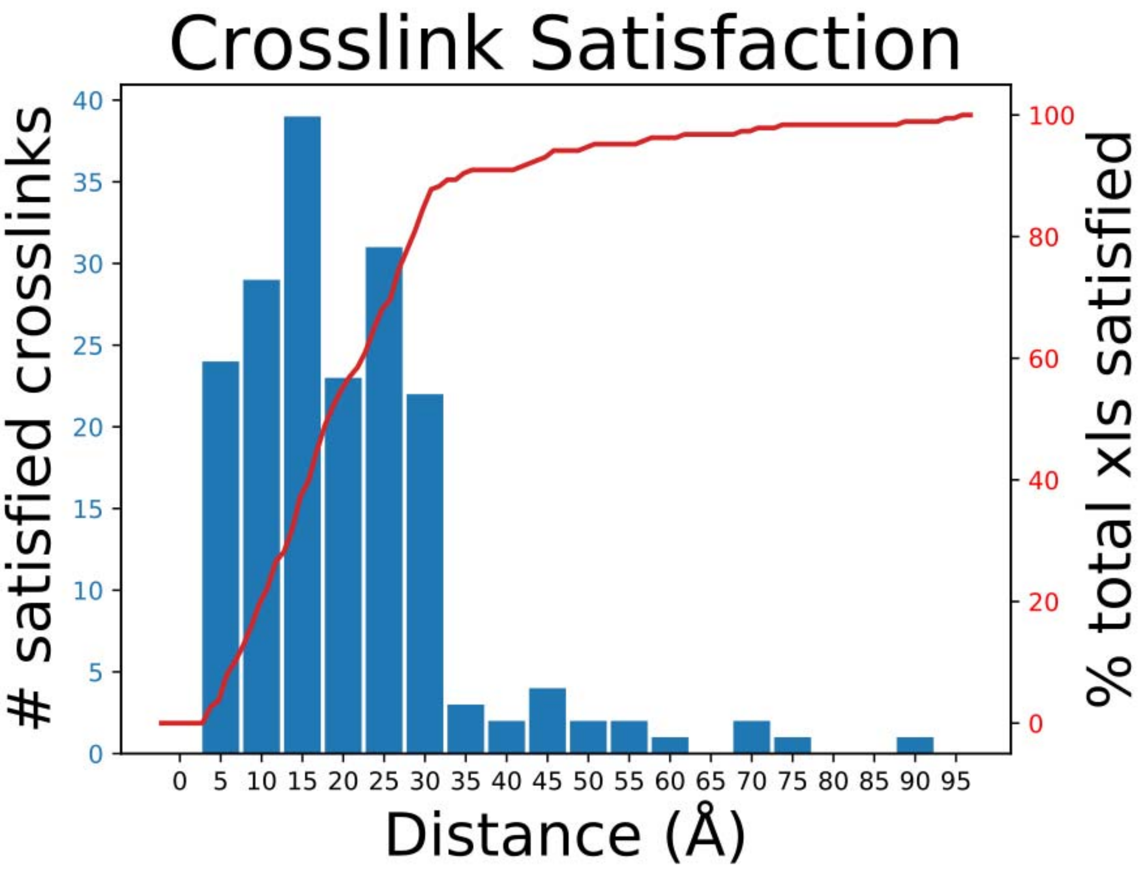

Figure S5 Evaluation of crosslink satisfaction across our final model. We see approximately 77% satisfaction of all crosslinks at a cutoff of 30Å which is acceptable given our BS3 crosslinker. The histogram in blue plots the number of crosslinks satisfied in 5 Å bins (0-5, 5-10, …) and is plotted against the left Y axis. The line plot in red is a continuous measure of the percent of the total number of crosslinks satisfied in our model (right Y axis) as a function of their evaluated distance in Å.

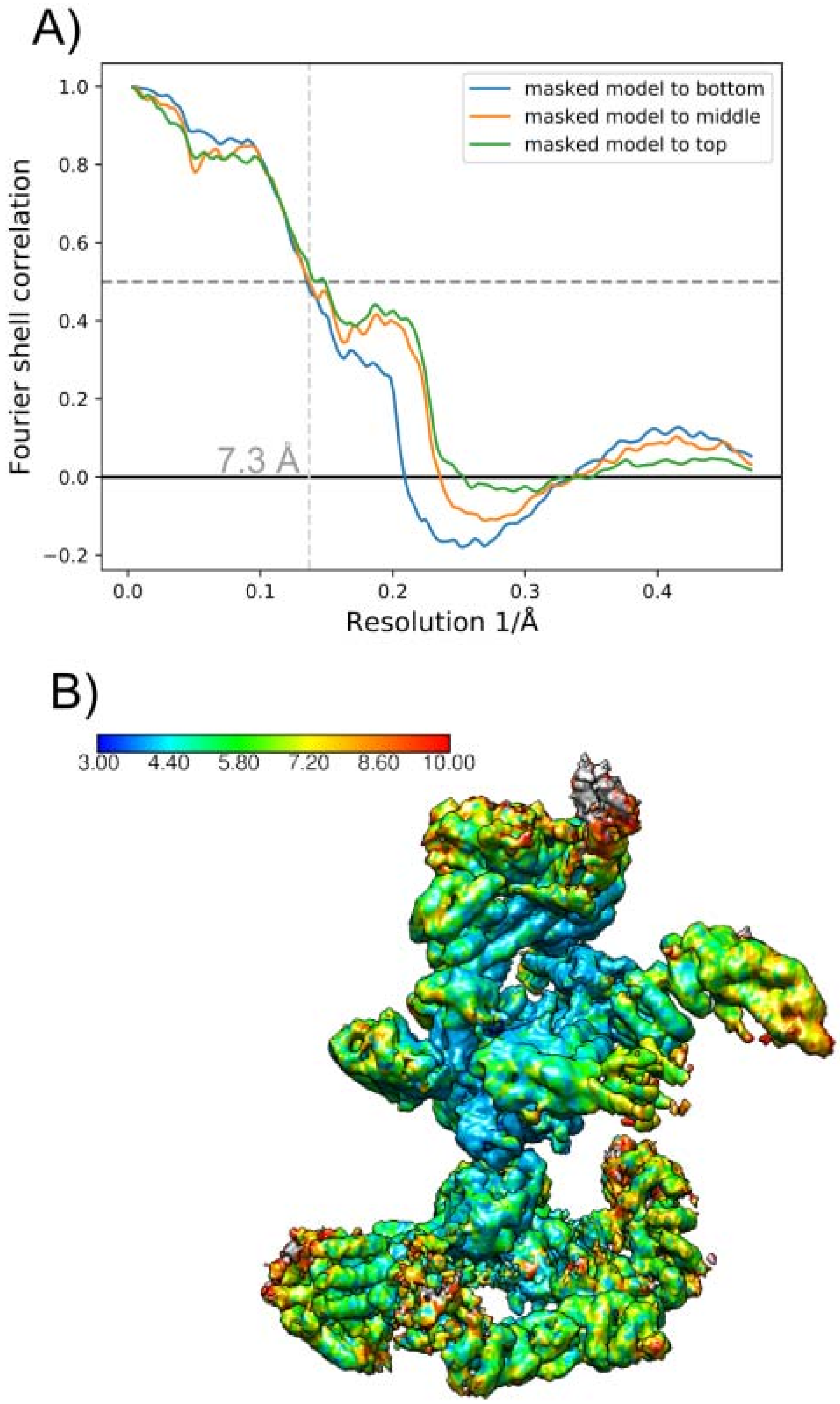

Figure S6 Evaluation of model from map. A) FSC plot of our model segmented to the three maps that generated the composite map used throughout modeling. B) Local resolution plot of the map used during modeling.
